# Effect of working characteristics and taught ergonomics on the prevalence of musculoskeletal disorders amongst dental students

**DOI:** 10.1186/1471-2474-14-118

**Published:** 2013-04-02

**Authors:** Saad A Khan, Kwai Yee Chew

**Affiliations:** 1 International Medical University, Jalan Jalil Perkasa 19, Bukit Jalil, Kuala Lumpur 57000, Malaysia

**Keywords:** Dental students, Musculoskeletal diseases, Work characteristics, Work environment, Ergonomics

## Abstract

**Background:**

Work-related musculoskeletal disorders are one of the main occupational health hazards affecting dental practitioners. This study was conducted to assess the prevalence of Work-related Musculoskeletal Disorder (WMSD) amongst dental students. Possible correlations with the working environment and ergonomics taught in Malaysian dental schools were also sought.

**Methods:**

Five dental schools in Malaysia participated in this cross-sectional study. A validated self-administered questionnaire was used to establish the point prevalence of WMSD in the dental students based on various body regions. The questionnaire also collected data regarding the working environment, clinical practice and the taught ergonomics of the students during their training years.

**Results:**

Out of five hundred and sixty eight dental students who participated in the study, 410 were in their clinical years whilst 158 were students in their non- clinical years. Ninety three percent of the clinical year students reported symptoms of WMSD in one or more body regions. Female students reported a significantly higher numbers of symptoms compared to male students. The neck (82%) and lower back (64%) were reported to have the highest prevalence of WMSD. Discomfort in the neck region was found to be associated with self-reported frequency of bending of the neck. A majority of students (92%) reported minimum participation in workshops related to ergonomics in dentistry and 77% were unfamiliar with treatment and remedies available in the case of WMSD.

**Conclusions:**

There was more WMSD seen in dental students who had started their clinical years. Neck and lower back are more injury prone areas and are at increased risk of developing musculoskeletal disorders. Theory and practice of ergonomics should be incorporated into the dental undergraduate curriculum.

## Background

Work-related musculoskeletal disorders (WMSD) are common amongst dental personnel, who work in a restricted field that makes high demands on vision, and requires them to sit in a static or awkward posture, use excessive force, as well as undertake precise repetitive hand and wrist movements [1,2]. Many studies have investigated the prevalence of WMSD amongst dentists. A systematic review on this topic focusing mainly on the pain experience found that the prevalence of WMSD ranged as high as 64% and 93% [3]. According to the World Health Organization (WHO) and the National Institute for Occupational Safety and Health (NIOSH), the causes of WMSD are multifactorial including not only workplace conditions and workplace exposures but also organizational, psychosocial and sociocultural variables, amongst others [4,5]. In our study the term WMSD refers to signs and symptoms arising due to series of micro traumas to bones, joints, ligaments, muscle tendons, blood vessels and nerves that accumulate and are intensified by work [6].

It is important to highlight this issue as WMSD in dentistry might contribute considerably to sick leave, reduced productivity and future possibility of leaving the profession at an early age [7-9]. It was reported that dentists who suffer musculoskeletal symptoms are more susceptible to neuro-circulatory disease, including varicose vein, postural defects, and flat (foot) feet with subsequent effects on their general health and well being [10].

It has been suggested that injuries caused by WMSD, or similar cumulative trauma disorders, can be reduced or prevented by applying ergonomics in dental equipment and instrument design [11]. Good ergonomic practices can prevent a number of WMSD conditions such as carpal tunnel syndrome. Adjusting the patient’s chair when accessing different quadrants, placing instruments and materials within easy reach, working with elbows lower than shoulders have been advised to improve posture in a clinical environment thus minimizing fatigue and the risk of developing WMSD [12].

On the other hand, some studies have shown that musculoskeletal pain was negatively correlated with years of experience [13,14]. It has been hypothesized that more experienced dentists learn to adjust their work posture to avoid such problems, or that those dentists with severe WMSD have left the profession [7]. Therefore, this suggests that even dental students can manifest early signs of WMSD during their years of training. These findings were supported by research that revealed that more than 70% of dental students experienced neck, shoulder and lower back pain as early as the third year of their dental training [14,15].

Previously much of the focus regarding WMSD has centered on dentists and dental hygienists, while WMSD prevalence amongst dental students has not been thoroughly addressed in the literature. An assessment of the WMSD amongst the dental students and the underlying factors associated with it, is required to more clearly elucidate the nature of this important issue for dental students [16].

Therefore, the main objective of the present study was to assess the point prevalence of WMSD among dental students in their clinical and non-clinical years. The secondary objective of the study was to correlate the prevalence of WMSD of clinical-year students with the work characteristics during their training years. In addition to this, students’ understanding of ergonomics and their capability for self-application during dental practice were also assessed. The aim of the study was to obtain this information which may improve the understanding of contributing risk factors thereby preventing early manifestation of WMSD.

## Methods

### Study design

This study used a descriptive analytical cross-sectional design. It was conducted during the period July 2011 – April 2012. The International Medical University Joint-Committee of the Research and the Ethics Committee (BDS I1/08(07)2011) approved this study.

### Study population

All Malaysian dental schools (n = 11) were invited to participate in this study except two dental schools^a^ where the students had not started their clinical years. Prior to data collection a request letter, copy of consent form and an information sheet were sent to respective dental schools to obtain approval and to provide better understanding of the research project. Amongst the 9 dental schools approached, 5 dental schools^b^ agreed to participate in the study.

Dental students’ representatives were assigned by the Deans to facilitate the administration of the survey. Data was collected between July 2011 and January 2012. Informed consent forms were obtained from participating students and confidentiality and anonymity were assured.

All dental students enrolled in the 5 participating dental schools were invited to participate in the survey. Students with previously diagnosed musculoskeletal disorders and students who were overly involved in sports or played musical instruments for more than 25 hours per week were excluded from the study. WMSD prevalence was assessed for all participating students. First and second year dental students were recruited into the study to act as a control group.

The questionnaire administered was adapted from a validated self-administered questionnaire developed and used in University of Connecticut, USA [17]. The questionnaire was given to three dental lecturers for informal feedback and pilot tested in 2 phases. The first phase was a panel discussion with 4 dentists. The questionnaire was modified based on the panel comments and feedback. The second phase was piloting the questionnaire with a group of 15 students. Content validity and face validity of the questionnaires were determined and indicated that they could be used for the purposes of the study. The pilot study results were as follows; 9 students reported lower back discomfort, 7 students reported neck discomfort, and 6 students reported hand and fingers discomfort. Lower shoulder and forearms discomfort were reported by 3 and 4 students respectively, while elbow discomfort was reported by 1 student.

The questionnaire comprised of four main sections: the first section addressed the demographics, the second section assessed the working environment and practicing characteristics, the third section addressed the taught ergonomics, while the fourth section assessed the prevalence of WMSD based on body regions. Outcomes were assessed with a series of questions adapted from the questionnaire. The primary outcome variable was the point prevalence of WMSDs.

Data were entered and analyzed using Statistical Package for Social Sciences (SPSS) Version 18. Chi-square tests and binary logistic regression were used to identify association between variables. Results with P-values of less than 0.05 were taken as statistically significant.

## Results

A total of 575 dental students participated in this study yielding a response rate of 81%. Four hundred and ten clinical year students participated in the study, while there were 158 non-clinical year students. The demographics of students enrolled in this study showed the characteristics are shown in (Table 1). Thirteen students were excluded due to medical problems, recent surgical procedures or because they engaged in more than 20 hours of sports per week. Reported medical problems included scoliosis, ligamental injuries, back problems due to history of a fall, prolapsed discs, carpal tunnel syndrome and poly-arthritis. All the missing data were checked. None of the questions had more than 5% missing values. The majority of questions had 5 or fewer missing values. An accuracy check of 20 randomly selected questionnaires, yielded no errors after data checking and cleaning procedures.

**Table 1:** Demographic characteristics of dental students

**Demographics**	**N**	**(%)**
Gender		
Male	148	26
Female	427	74
Type of University		
Public University	394	69
Private University	181	31
Ethnicity		
Malay	298	52
Chinese	255	44
Indians	10	2
Others	12	2
Right or left handedness		
Right Handed	530	92
Left Handed	45	8

The differences of discomfort due to WMSD between clinical and non clinical year students reported in various body regions was statistically significant at the level of (p < 0.05), (Table 2).

**Table 2:** Discomfort reported by clinical and non-clinical students in various regions of the body

**Discomfort**	**Non-clinical N (%)**	**Clinical N (%)**	**Chi square (<.001)**
Neck and Upper back	65 (41%)	336 (82%)	0.001
Lower Back	45 (28%)	264 (64%)	0.001
Hands and Fingers	13 (8%)	171 (42%)	0.001
Lower Shoulder	8 (5%)	108 (26%)	0.001
Forearms	4 (2%)	96 (23%)	0.001
Elbows	2 (1%)	53 (13%)	0.001

The secondary objective of the study was to correlate the WMSD findings amongst the clinical-year dental students with work characteristics. The findings reported here onwards reflect only the responses of the dental students in the clinical years.

Within the cohort of 410 clinical students 72% of female students reported symptoms of WMSD in one or more than one region of body while 20% of male students reported discomfort due to WMSD. Chi-square tests revealed a significant statistical difference between gender and reporting of WMSD as female students were more likely to have WMSD during their clinical years (p < 0.05).

### Working environment and its characteristics

Work environment and its characteristics were represented by four domains: Sitting position, instrument handling, use or otherwise of dental loupes and frequency of working hours. Three items addressed the sitting position, the use of a comfortable stool, adjusting the work stool based on height and back position and using the back support during practice. Four items addressed the handling of instruments, (including); reaching for instruments without strenuous movements, use of forceful movement to perform clinical work, performing clinical work with arms above shoulder height in addition to bending and twisting of the neck. The use of dental loupes was addressed in one item. Two items in the questionnaire assessed the number of working hours in the clinic and the number of hours using vibrating instrument. Chi-square and logistic regression analysis were conducted in order to gain a better understanding of the relationships between reported symptoms in various regions of the body and the four common working characteristics in the clinic.

1. Sitting position

In relation to sitting position of the students as part of their working environment, three variables were assessed: the use of a comfortable work stool, adjusting the work stool based on height and back position as well as having back support on the stool. Three hundred and nineteen (78%) students indicated that the stool they used was comfortable. Adjusting the height of the chair was found to be a common practice among the students as a total of 353 (86%) students reported that they often adjusted the chair prior to their work. One hundred and forty (34%) and 38 (9%) students reported that they “often” and “very often” sat with their back supported while 176 (43%) reported “seldom” and 56 (14%) reported “never” to the question about adjusting the back support.

There was a significant association between the presence of lower back pain and the sitting position using a comfortable work stool (p < 0.05) and having a back support (p < 0.05). This means that students who used a comfortable work stool with a back support were less likely to have lower back pain. However, there was no statistically significant association between lower back pain and adjusting the height of the chair.

2. Instrument handling:

Regarding the handling of instruments, four working characteristics were assessed: whether four handed dentistry is facilitated at the school, whether instruments were within hand reach so avoiding the need for strenuous movements, work being done with arms above shoulder height and finally, using forceful movements to perform clinical work.

Three hundred and ninety nine (98%) students had four-handed dentistry facilities at their respective dental schools. Statistical analysis showed that students who practiced four-handed dentistry at their dental school were less likely to report symptoms in both elbow (p < 0.05) and forearm (p < 0.05). There was no association with symptoms in other body regions.

The ability to reach the instrument without making strenuous movement was reported by 287 (70%) students. Statistical analysis revealed no association between approaching the instruments easily and WMSD symptoms in the lower shoulder, forearm and elbow.

Work done in clinics with arms above shoulder height and forceful movement of arms while working were found to be statistically associated with WMSD symptoms in the lower shoulder and forearm while no statistically significant association was detected between these two variables and WMSD symptoms in the elbow (Table 3).

**Table 3:** Working characteristics of clinical year students and its association with WMSD symptoms in lower shoulder, forearm and elbow

	**Very often N (%)**	**Often N (%)**	**Seldom N (%)**	**Never N (%)**	* Lower shoulder	*Forearm	*Elbow
Work done in clinics with arms above shoulder height	26 (6%)	56 (14%)	174 (42%)	154 (38%)	0.002**	0.001**	0.057
Forceful movements with arms	41 (10%)	92 (22%)	253 (62%)	23 (6%)	0.001**	0.001**	0.054

* Association between WMSD symptoms in these regions and the working characteristics.

**Significant at p-value < 0.05.

With regard to bending and twisting of the neck, two hundred and three (50%) and 72 (18%) students reported that they “often” and “very often” bent or twisted their neck when treating patients. Discomfort at the neck and upper back region was reported by 336 students (82%). Statistical analysis revealed a significant association (p < 0.05) between neck and upper back discomfort and bending or twisting neck movements during clinical work.

3. Dental loupes:

Out of 410 clinical students, 331 (81%) did not use dental loupes. No statistical significance was found between use of dental loupes and prevalence of discomfort in the neck and upper back.

4. Working hours:

Two variables in the form of continuous data were analyzed to identify any association between number of working hours in the clinic and frequency of using vibrating instruments and discomfort reported in body region. Prior to logistic regression, missing values for hours per week were replaced with the sample median. Different missing value strategies are likely to yield similar results when the total number of missing values is less than 5% of potential responses.

Binary logistic regression was used in this study where finger and hand discomfort was the dichotomous criterion variable and the predictor variables were the number of working hours and the number of hours using vibrating instruments.

Calibration of the logistic model was assessed using the Hosmer–Lemeshow goodness–of–fit test to evaluate the discrepancy between observed and expected results of hand and finger discomfort. The test showed that the model was well calibrated with P = .172 indicating that there is no large discrepancy between observed and expected results and indicating also that the data fit the model well when the clinical hours were used as a predictor for hand and finger discomfort.

Logistic regression analysis indicated that the number of clinical working hours per week were statistically significant risk factors for hand and finger discomfort, (Table 4). Students with increased number of working hours per week were much more likely to report discomfort in hands and fingers (OR: 12.667, 95% CI: 1.17-221.8). Interestingly, the number of hours using vibrating instruments was not associated with the hand and finger discomfort (OR: 0.724, 95% CI: 0.301-1.506).

**Table 4:** Logistic regression predicting likelihood of developing discomfort in body region

	**Correlate**	**OR**	**95% CI**	**P-value**
Hand and finger discomfort	Number of clinic working hours	12.667	1.17-221.8	.000
	Number of hours using vibrating instruments	0.724	0.301-1.506	.01

The fourth part of the questionnaire addressed the taught ergonomics and prevention of WMSD. Responses in relation to this part are shown in Table 5. Ninety three percent of the students had never attended any hands-on training and/or workshop on preventing WMSD at their dental school and only 20% reported exercising following clinical work, which is the key factor for preventing the initiation of musculoskeletal disorder. Eighty percent of the students showed willingness to be evaluated for WMSD.

**Table 5:** Reporting of taught ergonomics and prevention by clinical students

	**Yes (n) %**	**No (n) %**
Is Ergonomics a taught subject at your Dental School	170 (42%)	240 (58%)
Have you ever attended a workshop on preventing musculoskeletal disorders (MSD) at your Dental school?	30 (7%)	380 (93%)
Are you familiar with preventive techniques to decrease the possible risk of having MSD?	208 (51%)	202 (49%)
Are you familiar with remedies/treatment options once you are subjected to an MSD problem?	94 (23%)	316 (77%)
After finishing clinical practice, do you perform stretching exercise?	84 (20%)	326 (80%)
Would you like to be evaluated for MSD symptoms at your Dental school?	82 (20%)	328 (80%)

## Discussion

Work-related musculoskeletal disorders are one of the main occupational health hazards affecting dental practitioners and dental students [18]. This study highlights and supports the established fact that WMSD is a major concern for dental students during their training years [12,19].

The results of the current study revealed a significant difference in the prevalence of discomfort and WMSD symptoms between dental students in their clinical and non-clinical years. This can be attributed to difference in the nature of work, practicing pattern and working hours between clinical and non-clinical years of dental education. This finding was in agreement with that of another study conducted to investigate the distribution and severity of musculoskeletal pain among dental students. The authors reported increase in pain prevalence with the number of years spent in the dental school and this was more related to students in clinical years acquiring clinical skills and providing routine dental procedures [14].

The study identified three body regions with the highest prevalence of WMSD amongst students in clinical and non-clinical years. Neck, upper back and lower back regions showed the highest prevalence of discomfort in comparison to other body regions. Dajparatham, De Carvalho, Rising and colleagues reported that cervical and dorsal regions are common pain regions of WMSD manifestation and they are generally symptomatic injury prone areas [14,16,19]. In a study conducted to establish basic epidemiological data on chronic pain the prevalence of neck , upper back and lower back pain was reported to be the highest amongst all body regions [20]. These regions were considered injury prone areas as they are more mobile within the lumbar and cervical curves and can be affected more easily [21].

The difference in pain reporting between clinical and non-clinical students with a higher incidence during the clinical years can be attributed to their practice time that is significantly more than the dental students in the non clinical years in addition to the nature of this clinical exposure [14].

Gender is considered a potential risk factor for developing WMSD. Results of this study showed consistency with previous studies as female dental students showed a higher prevalence of WMSD symptoms than males. The literature attributed these results to the smaller body habitus and lower muscle tone of females in addition to higher stress levels and psychosocial factors affecting females [22,23].

Working environment and its characteristics were considered as major factors affecting the prevalence of WMSD [24]. This study identified 4 main areas related to working environment and assessed them in relation to the reporting of WMSD in different body regions. The areas identified were the sitting position, handling of dental instruments, use of dental loupes and working hours.

Sitting position was identified as one of the major working factors that can contribute to WMSD. It was reported that dental practitioners spend a mean of 44 hours per week in their practice [25] and they spend about 78 percent of their working time seated [26]. In regards to the dental stool it is recommended to obtain a stool that offers neutral back, neck and shoulder support for optimal posture and possesses an adjustable height and tilt [27,28].

Sitting position is represented in this study by having a comfortable work stool, back support while sitting and adjusting the stool-height and back position. Results of this study found that having a comfortable dental chair with a back support are likely to decrease the prevalence of lower back discomfort. However, adjusting the chair height was found to have no effect on the prevalence of lower back discomfort.

Reaching for instruments and instrument handling are normal procedures in dental practice. Awkward posture, repetitive movements, and inappropriate force contributes to the prevalence of WMSD [29]. Within instrument handling the questionnaire included 5 items; reachability of the instruments without strenuous movement, use of forceful movement with the arm, performing clinical work with the arm above the shoulder height, four-handed dentistry and bending and twisting the neck during clinical work.

Forearm and lower shoulder discomfort were found to be significantly associated with working while arms were above the shoulder and with forceful arm movements. This finding was supported in the literature as working with arms above the shoulder was reported as a predisposing factor to trapezius myalgia and rotator cuff impingement; the first is associated with pain, tenderness and muscle spasms while the later is associated with pain in shoulder on overhead reaching or sustained arm elevation [30].

Four-handed dentistry is accepted as part of the current dental practice, and defined as an ergonomic chairside work arrangement performed by a well –trained dental team in an organized manner. The overall concept provides a synergistic approach to dental practice that provides more efficient delivery of dental care and increased productivity [31]. Results of this study revealed wide spread use of four –handed dentistry amongst the dental students in Malaysia as 98% of the students reported working with an assistant. This study supported the importance of four-handed dentistry in relation to elbow and forearm discomfort. As the use of four-handed dentistry was significantly associated with lower prevalence of both. This maybe attributed to the ease of handling and manipulating instruments in four-handed dentistry and less effort made by the dentist in reaching and handling instruments. In relation to other body regions four –handed dentistry showed no association with the discomfort encountered in these parts. However, another study suggested that it has contributed to an increase in prolonged static posture among operators [30].

On the other hand, bending and twisting of the neck during clinical work was found to be highly associated with neck and upper-back discomfort and this finding was consistent in the literature as neck extension, flexion and rotation were identified as possible risk factors contributing to neck discomfort and pain [32]. A literature search revealed that forward head posture and neck position may predispose to tension neck syndrome with associated symptoms of pain, stiffness, and muscle spasm with referring pain between shoulder blades and these findings were in agreement with the findings of this study [30].

Results of this study showed that the use of dental loupes was not a common practice amongst students in Malaysian dental schools. Only 19% of the students reported using dental loupes during clinical work. The findings of this study were in contrast to those reported by James and colleagues as they stated that the use of dental loupes is becoming an accepted norm amongst undergraduate population [33]. The limited use of dental loupes can be attributed to their relative high cost within this region. The literature supports the use of dental loupes as they can enhance visualization of fine details and maintenance of good ergonomic posture [34].

Results of this study found that students who work for longer hours in clinic were 12 times more likely to report discomfort in one or more body region. Additionally, discomfort in hands and fingers only showed a weak correlation with the number of hours using vibrating instruments. Nevertheless, handling of vibrating instruments was found to be associated with nerve trapping, early arthrosis and Reynolds’s syndrome resulting in subsequent hand and finger pain. [35] Therefore, considerations such as taking micro-breaks of 50 seconds in between treating patients, lessening the hours using vibrating instruments and even finger exercise would be useful in reducing the muscle strain and optimizing the strength capacity of the operator [36].

Ergonomics is the science of designing jobs, equipment and workplaces to fit workers. Proper ergonomic design is necessary to prevent repetitive strain injuries, which can develop over time and can lead to long-term disability [37]. In this study, 58% of the students indicated that ergonomics had not been a taught subject in their curriculum while 93% of students had never attended a workshop on preventing MSD at their dental school. This coincides with the findings of two other studies where the first study indicated that most of the dental students had not attended courses on WMSD or ergonomics in the course of their dental education, [38] and the other (later) concluded that knowledge of ergonomics, postural requirement and their clinical application was not satisfactory among the dental students surveyed [39].

In regards to the prevention and management aspect of WMSD, as many as 77% of students were not familiar with remedies or treatment options should they be subjected to an MSD problem. While fifty one percent of the students claimed that they were familiar with the prevention of WMSD only 20% of them reported exercising following clinical work. This finding implies that there might be a gap between theory and practice when it comes to WMSD prevention. Therefore, more emphasis should be put on the acquisition of ergonomics knowledge during the early years of dental programs in order to allow students to apply their theoretical ergonomics knowledge to their clinical practice and help preventing deleterious habit formation [40].

The last item of the questionnaire addressed whether the students’ would like to be evaluated for WMSD symptoms or not and it was found that 80% of the students would like to be evaluated. This finding indicates that dental students are concerned about the WMSD symptoms.

The main limitation of the study is that the cross sectional design used was not able to establish a temporal relationship between prevalence of discomfort (WMSD) and work characteristics. Self-reporting by the dental students is another relationship which may affect the factors -including inability to record all incidents of WMSDs, in addition to over or under estimation of the pain and the related injuries. However the use of well-structured questionnaires as a study tool was useful for identifying the prevalence of WMSD amongst the dental students.

## Conclusion and recommendations

Overall, the study demonstrates that WMSD may represent a significant burden for dental students in Malaysia. The reporting of musculoskeletal symptoms by dental students as early as the first year of the dental program suggests that ergonomics should be covered and taught as part of the dental curriculum to reduce risks of WMSD in the future. Therefore, ergonomics improvements, health promotion and institutional interventions are needed for reducing the risks for WMSD.

To reduce discomfort in the neck and upper back it is highly recommended that working distance should be maintained for optimal posture, with shoulders relaxed and elbows closed to the sides. It is important that 20 degree or less flexion of neck is maintained- this will avoid the operator to hunch over the patient. Dental loupes with magnification of 2x are sufficient to visualize the working field details. Before starting any clinical work, the operator must adjust the arm rest which will improve elbow support and decrease neck and shoulder fatigue. Discomfort in the lower back can be prevented by using a saddle type operator stool with lumbar support. This is to maintain the natural lower back curvature which also allows to the operator to stay closer to the patient. The lumbar support should always stay in contact with the operator’s back.

It is recommended to conduct more research through observational studies, physical examination and assessment. It is equally important to develop intervention programs to address the students’ knowledge and practice of ergonomics.

## Endnotes

^a^Manipal College of Dental Science and MAHSA University College.

^b^Universiti Kebangsaan Malaysia (UKM), International Medical University (IMU), Universiti Sains Malaysia (USM), Universiti Malaya (UM) and Universiti Teknologi MARA (UiTM).

## Competing interests

Conflict of interest: none, Professor Tim Morse has provided the questionnaire and has given the permission to authors use for this study.

## Authors’ contributions

SAK initiated the study and prepared the proposal. KYC was responsible for data collection and data entry. Both authors did the data analysis, interpretation and write up of discussion. Both authors read and approved the manuscript.

## Pre-publication history

The pre-publication history for this paper can be accessed here:

http://www.biomedcentral.com/1471-2474/14/118/prepub
